# Dietary flavonoid intake and cardiovascular risk: a population-based cohort study

**DOI:** 10.1186/s12967-015-0573-2

**Published:** 2015-07-08

**Authors:** Valentina Ponzo, Ilaria Goitre, Maurizio Fadda, Roberto Gambino, Antonella De Francesco, Laura Soldati, Luigi Gentile, Paola Magistroni, Maurizio Cassader, Simona Bo

**Affiliations:** Department of Medical Sciences, University of Turin, Corso Dogliotti 14, 10126 Turin, Italy; Unit of Clinical Nutrition, “Città della Salute e della Scienza” Hospital of Turin, Turin, Italy; Department of Health Sciences, University of Milan, Milan, Italy; Diabetic Clinic, Hospital of Asti, Asti, Italy; Immunogenetics and Transplant Biology, “Città della Salute e della Scienza” Hospital of Turin, Turin, Italy

**Keywords:** All-cause mortality, Cardiovascular risk, Cardiovascular mortality, Flavonoids

## Abstract

**Background:**

The cardio-protective effects of flavonoids are still controversial; many studies referred to the benefits of specific foods, such as soy, cocoa, tea. A population-based cohort of middle-aged adults, coming from a semi-rural area where the consumption of those foods is almost negligible, was studied.

**Aims:**

The primary objective was establishing if flavonoid intake was inversely associated with the cardiovascular (CV) risk evaluated after 12-year follow-up; the associations between flavonoid intake and CV incidence and mortality and all-cause mortality were also evaluated.

**Methods:**

In 2001–2003, a cohort of 1,658 individuals completed a validated food-frequency questionnaire. Anthropometric, laboratory measurements, medical history and the vital status were collected at baseline and during 2014. The CV risk was estimated with the Framingham risk score.

**Results:**

Individuals with the lowest tertile of flavonoid intake showed a worse metabolic pattern and less healthy lifestyle habits. The 2014 CV risk score and the increase in the risk score from baseline were significantly higher with the lowest intake of total and all subclasses of flavonoids, but isoflavones, in a multiple regression model. During follow-up, 125 CV events and 220 deaths (84 of which due to CV causes) occurred. CV non-fatal events were less frequent in individuals with higher flavonoid intake (HR = 0.64; 95%CI 0.42–1.00 and HR = 0.46; 95%CI 0.28–0.75 for the second and third tertiles, respectively) in Cox-regression models, after multiple adjustments. All subclasses of flavonoids, but flavones and isoflavones, were inversely correlated with incident CV events, with HRs ranging from 0.42 (flavan-3-ols) to 0.56 (anthocyanidins). Being in the third tertile of flavan-3-ols (HR = 0.68; 95% CI 0.48–0.96), anthocyanidins (HR = 0.66; 95% CI 0.46–0.95) and flavanones (HR = 0.59; 95% CI 0.40–0.85) was inversely associated with all-cause mortality. Total and subclasses of flavonoids were not significantly associated with the risk of CV mortality.

**Conclusions:**

Flavonoid intake was inversely associated with CV risk, CV non-fatal events and all-cause mortality in a cohort with a low consumption of soy, tea and cocoa, which are typically viewed as the foods responsible for flavonoid-related benefits.

## Background

Flavonoids are a group of plant metabolites widely distributed in the plant kingdom with antioxidant properties, which can be classified into seven subgroups based on their chemical structure: flavanones, flavones, flavonols, flavan-3-ols, anthocyanidins, isoflavones and proanthocyanidins [1]. These compounds are present in small quantities in fruits, vegetables, tea, wine, nuts, seeds, herbs, spices, cocoa, soybean [2–4].

A wide spectrum of health benefits, such as antioxidant, anti-inflammatory, antibacterial, antithrombotic, anti-carcinogenetic properties have been reported for flavonoids [5].

Many epidemiological studies have reported inverse associations between the total flavonoid intake or the intake of specific classes of flavonoids and the incidence or mortality for cardiovascular (CV) diseases [6–20]. However, not all studies agreed about the cardio-protective effects of these compounds [21–27]. Many studies referred to specific foods, which are the main sources of flavonoids in different populations, such as tea [13, 28, 29], cocoa [28, 30], soy [17].

We have studied a population-based cohort of middle-aged adults, coming from a semi-rural area, where the consumption of some flavonoid-rich foods, such as cocoa and soybean is almost negligible, while the most important sources of flavonoids are fruits and red wine.

The primary objective of this study was establishing if the consumption of flavonoids was inversely associated with the CV risk evaluated after 12-year follow-up; the secondary aims were evaluating the associations between flavonoid intake and CV incidence, CV mortality, and all-cause mortality in our cohort.

## Methods

All the Caucasian patients (*n* = 1,877), aged 45–64 years, of six family physicians were invited to participate in a metabolic screening in 2001–2003. These subjects were representative of the Local Health Units of the province of Asti (northwestern Italy) [31]. Exclusion criteria were: inability to go to the office of the family physician and to give the informed consent.

Of these, 1,658 (88.3%) subjects gave their written informed consent to participate and 219 patients declined. Both the participants and non-participants were similar to the resident population of a corresponding age range with respect to the percentage of males, level of education, prevalence of known diabetes, and residence in a rural area [31]. The study was approved by the local ethics committee. All procedures conformed to the principles of the Helsinki Declaration.

### Methods

In the morning and after fasting, weight, height, waist circumference, and blood pressure were measured in the office of the family physicians. Glucose, insulin, total cholesterol, HDL-cholesterol, triglyceride, uric acid and high-sensitivity C-reactive protein (CRP) levels were determined. If the serum glucose value was ≥110 mg/dl, a second fasting glucose determination was performed. Two blood pressure measurements were performed with mercury sphygmomanometers and the appropriate cuff sizes after a 10-min rest in the sitting position, and the values reported are the means of the two measurements. The waist circumference was measured by a plastic tape meter at the level of the umbilicus. The measurements were performed by trained physicians holding a grant.

Patients completed the Minnesota Leisure Time Physical Activity questionnaire [32]. The physical activity level was calculated as the product of the duration and frequency of each activity (in hours/week), weighted by an estimate of the metabolic equivalent of the activity and summed for the activities performed.

From January to November 2014, the patients were submitted to a blood sample analysis and a follow-up visit by their family physicians. Information on the vital status of each patient and the causes of death of those who died was collected from the demographic files of the town of residence or death.

The laboratory methods have been described previously [31, 33]. All samples were run blindly.

### Ascertainment of flavonoid intake

The semi-quantitative food-frequency questionnaire used in the EPIC (European Prospective Investigation into Cancer and Nutrition) studies was used for all subjects [34]. It assessed average frequency and portion intake of 148 foods consumed during the 12 months before the enrolment. For each food item, the participants had to mark if the food or dish was consumed or not during the previous year. For all food items consumed, the subjects should select their typical portion size with the help of photographs, the consumption frequency and the time period (day, week, month or year), which suited them best. Questions about the type of fat for cooking were also included. This tool has been previously validated [34]. A dietician, blinded to the study details, checked all questionnaires for completeness, internal coherence, and plausibility. In case of uncertain answers, the patients were interviewed by the dietician.

Each nutrient was adjusted for total energy, using the residual method [35]. The reliability of the reported energy intake was assessed by calculating the ratio of estimated energy intake to predicted basal metabolic rate, using age- and sex-specific formulas derived by Schofield [36]. Subjects with a ratio <0.88 were classified as under-reporters [37].

Dietary intake of total and subclasses of flavonoids were estimated by using the latest detailed food composition tables published by the US Department of Agriculture (USDA) on the seven major classes of flavonoids [2–4] and extended with information from an European database [38]. Merging of the databases gave a single data-file. Flavonoid intake was computed by multiplying the specific flavonoid content of the serving of each food item (mg aglicone equivalent/100 g food) by the daily consumption (g/day) of the selected food item. Estimated total intake of individual flavonoids was the sum of individual flavonoid intakes from all food sources reported in the questionnaire. Total flavonoid intake was calculated by summing up the seven subclasses (flavanones, flavones, flavonols, flavan-3-ols, anthocyanidins, isoflavones and proanthocyanidins), and were expressed as mg/day aglycones.

The contribution of each food to the total intake of subgroup and total flavonoids was calculated as a percentage; single foods were then grouped into large categories.

All flavonoid subgroups that were estimated, their respective compounds, and the main food sources are shown in Table 1.

**Table 1:** Flavonoid classes and compounds, and respective dietary intakes and main food sources in the whole cohort

	Compounds	Median intake (mg/day)	Sources (%)
Total flavonoids		251.0	Fruits (38) Red wine (25) Vegetables (5) Tea (5)
Proanthocyanids	Dimers, Trimers, 4-6mers, 7-10mers, polymers of flavon-3-ols or flavanols	96.1	Fruits (50) Red wine (23) Legumes (6)
Flavan-3-ols	(−)-Epicatechin (−)-Epicatechin 3-gallate (−)-Epigallocatechin (−)-Epigallocatechin 3-gallate (+)-Catechin (+)-Gallocatechin Theaflavin Theaflavin-3, 3′-digallate Theaflavin-3′-gallate Theaflavin-3-gallate Thearubigins	50.4	Fruits (26) Tea (21) Red wine (19)
Anthocyanidins	Cyanidin Delphinidin Malvidin Pelargonidin Peonidin Petunidin	32.9	Red wine (53) Vegetables (17) Fruits (11)
Flavanones	Eriodictyol Hesperetin Naringenin	24.2	Fruits (71) Red wine (12)
Flavonols	Isorhamnetin Kaempferol Myricetin Quercetin	14.4	Vegetables (34) Red wine (14) Fruits (13)
Flavones	Apigenin Luteolin	1.2	Vegetables (51) Red wine (18) Fruits (8)
Isoflavones	Daidzein Genistein Glycitein	0.7	Legumes (90)

Sources contributing to ≥5% of the intake.

### Definitions

Alcohol intake was assessed by multiplying the mean daily consumption of each beverage by its ethanol content, to give grams of alcohol/day. Moderate and heavy drinkers were considered in the case of consumption of ≤30 and >30 g/day alcohol, respectively, in line with Italian guidelines [39].

Diabetes mellitus was defined according to published recommendations [40]. Estrogen use included both contraceptive medications or estrogen replacement therapy. The use of nutritional supplements was infrequent in this cohort and was limited to multivitamin, iron, calcium or, less frequently, magnesium.

The CV risk score was estimated with the Framingham risk score [41]. The diagnosis of CV disease was based on documented events that were recorded by the family physician (i.e. angina, previous myocardial infarction, coronary artery by-pass graft or another invasive procedure to treat coronary artery disease, transient ischemic attack, stroke, gangrene, amputation, vascular surgery, intermittent claudication, absent foot pulses and abnormal brachial and posterior tibial blood pressure using Doppler techniques).

The underlying cause of death was available for all the deceased patients and was derived from the death certificate and coded according to the ICD-9 (International Classification of Diseases, Ninth Revision). Deaths due to CV diseases corresponded to ICD codes 410–414 (coronary artery diseases), 430–438 (strokes), 440 (peripheral artery diseases) and other ICD codes between 390–459 and 798.1 (other CV diseases).

### Statistical analyses

Considering a type I error of 0.05 and a type II error of 0.90, a minimum of 83 subjects were needed for each tertile to detect a 10% difference in the CV scores between the tertiles of total flavonoid intakes.

Dietary total flavonoid intakes of the cohort were divided into tertiles, separately per sex. Cut-off points were 191.5, 401.2 and 138.3, 322.3 mg/day, respectively for men and women.

The distributions of flavonoid intake, fasting insulin, triglycerides, CRP values were skewed. The characteristics of the cohort according to the tertiles of flavonoid intakes were analyzed by ANOVA, Kruskal–Wallis tests (for not-normally distributed variables) or the χ^2^ test, as appropriate.

A multiple regression was performed to assess the association between the 2014 CV score and the variations from baseline to follow-up (values at 2014 minus values at baseline) in the CV risk score, and the tertiles of flavonoid intakes, after adjusting for BMI, education (primary/secondary/university), living in a rural area, METs (hour/week), alcohol intake (g/day), history of CV diseases, values of fasting glucose, log-CRP, fiber, and saturated fatty acid intakes. We did not include age, sex, total and HDL-cholesterol, smoking habits and blood pressure values, because these variables were included in the CV score calculation, to avoid over-controlling. However, when we controlled for these variables, results were not significantly different.

The relationships between tertiles of flavonoid intakes and all-cause mortality and CV mortality and incidence were assessed by estimating the hazard ratio (HR) and its 95% confidence intervals (CI) in Cox regression models, adjusted for age, sex, BMI, education, living in a rural area, METs (hour/week), alcohol, fiber, and saturated fatty acid intakes, smoking, values of systolic and diastolic blood pressure, total and HDL-cholesterol, fasting glucose, CRP, statin and aspirin use.

In all these analyses, individuals in the first (lower) tertile of flavonoid intakes were considered as the reference group, and the other groups were introduced as dummy variables (IBM SPSS Statistical Software Version 22).

## Results

Out of 1,658 subjects, 138 (8.3%) resulted under-reporters. Among the tertiles of flavonoid intake, the percentage of under-reporters did not differ (8.2, 8.5 and 8.3% in the first, second and third tertiles, respectively).

Mean and median intake of total flavonoids were 320 and 251 mg/day, respectively (Table 1).Pearson correlations between flavonoids ranged from weak (r = 0.04 for flavan-3-ols with isoflavones) to high (r = 0.80 for flavan-3-ols with proanthocyanids).

Descriptive characteristics of the cohort by tertiles of flavonoid intakes are shown in Table 2.

**Table 2:** Baseline characteristics by tertiles of flavonoid intake (the first the lower; the third, the higher)

	First tertile		Second tertile		Third tertile		P
Number	552		551		555		
Median intake (mg/day)	89.0		251.4		532.3		
Current smoking (%)	28.4		25.7		17.5		<0.001
Males (%)	47.1		46.8		47.2		0.99
Living in a rural area (%)	36.8		36.8		47.4		<0.001
Alcohol							
Alcohol abstainers (%)	54.9		41.0		35.7		
Moderate alcohol drinking (%)	31.5		41.4		38.6		
Heavy alcohol drinking (%)	13.6		17.6		25.8		<0.001
Education							
Primary school (%)	78.6		69.9		75.3		
Secondary school (%)	14.5		21.8		17.5		
University (%)	6.9		8.4		7.2		0.02
History of hypertension (%)	56.5		47.0		50.5		0.006
History of diabetes mellitus (%)	8.5		4.0		4.5		0.002
History of CV disease (%)	6.5		5.3		5.1		0.52
Estrogen use (%)	4.2		5.1		5.1		0.72
Supplements use (%)	3.3		3.3		3.4		0.98
Statin use (%)	3.8		3.8		4.7		0.70
Aspirin use (%)	6.0		6.0		4.1		0.29

	Mean	SD	Mean	SD	Mean	SD	
METS (h/week)	20.5	9.4	21.7	9.5	21.9	9.6	0.04
Age (years)	54.8	5.8	54.3	5.5	54.6	5.5	0.37
BMI (kg/m^2^)	27.2	5.3	26.3	4.3	26.3	4.3	<0.001
Waist circumference (cm)	92.9	13.0	90.2	12.4	90.9	13.4	0.002
Total caloric intake (kcal/day)	1,917.0	722.7	2,142.7	583.2	2,149.3	667.1	<0.001
CHO intake (% total kcal)	47.6	7.6	48.4	6.8	49.5	6.8	<0.001
Total fat intake (kcal/day)	35.6	6.1	35.2	5.9	34.7	5.7	0.03
Saturated fat (% total kcal)	12.3	3.4	12.1	2.8	11.6	3.0	0.001
Polyunsaturated fat (% total kcal)	4.3	1.3	4.3	1.6	4.3	1.4	0.95
Fiber intake (g/day)	16.5	7.3	22.4	8.3	23.3	10.2	<0.001
Beta-carotene (µg/day)	2,768.7	1,658.9	3,571.5	1,840.2	3,914.8	2,292.4	<0.001
Vitamin C (mg/day)	134.9	44.5	142.4	55.0	142.8	49.4	0.01
Vitamin E (mg/day)	8.1	3.0	8.1	2.3	8.2	2.6	0.62
Systolic blood pressure (mmHg)	135.4	16.6	132.3	15.0	133.3	16.0	0.007
Diastolic blood pressure (mmHg)	84.3	9.1	82.6	9.2	82.9	9.6	0.005
Fasting glucose (mg/dl)	109.0	38.7	102.2	24.3	103.5	26.6	<0.001
Fasting insulin	9.3	6.1	8.3	3.9	8.2	4.4	<0.001*
Total cholesterol	217.9	39.5	215.2	40.0	217.7	42.2	0.46
HDL cholesterol	57.9	12.5	60.9	13.1	62.4	14.1	<0.001
Triglycerides	149.1	82.9	131.7	99.9	137.0	92.4	<0.001*
CRP (mg/l)	3.3	7.0	2.4	4.7	2.3	5.2	<0.001*
Uric acid	3.4	1.0	3.3	1.1	3.3	1.0	0.14
CV risk score	12.6	8.3	10.6	6.8	10.5	7.1	<0.001

*CHO* carbohydrates, *CRP* C-reactive protein, *CV* cardiovascular.

*Kruskall–Wallis test for not-normally distributed variables.

In the lowest tertile, there was a higher percentage of smokers, alcohol abstainers, less educated individuals, hypertensive and diabetic patients (Table 2). On the other hand, subjects with the highest flavonoid intake were more frequently heavy drinkers living in a rural area, were more physically active, ate more calories, fiber and antioxidant vitamins, and less total fat and saturated fat. In individuals within the lowest tertile, the metabolic pattern was significantly worse, CRP values increased, and the CV risk score higher.

The 2014 CV risk score was significantly increased in the individuals with the lowest intake of total flavonoids and their subclasses, with the exception of isoflavones (Table 3). Similarly, after a mean 12-year follow-up, the difference in the scores (2014 score minus baseline score) was higher in those subjects. In a multiple regression models, being in the third (higher) tertile of flavonoid intake was inversely associated with the 2014 CV score and with change in score values from baseline to follow-up, after adjusting for BMI, education, living in a rural area, METs (hour/week), history of CV diseases, values of fasting glucose, log-CRP, alcohol, fiber, and saturated fatty acid intakes.

**Table 3:** CV risk score by tertiles of flavonoid intake (the first the lower; the third, the higher) in a multiple regression model

	First tertile		Second tertile			Third tertile		
Total flavonoids	Mean	SD	Mean	SD		Mean	SD	P
2014 CV risk score	28.8	15.4	25.3	12.6		23.8	10.7	<0.001
	**Β**		**β**	**95% CI**	**P**	**Β**	**95% CI**	**P**
Model 1	Reference		−2.58	−4.07 −1.09	<0.001	−4.36	−5.85 −2.87	<0.001
Model 2	Reference		−1.27	−2.76 0.22	0.10	−2.69	−4.22 −1.16	<0.001

	Mean	SD	Mean	SD		Mean	SD	P
Changes in CV risk score	16.2	10.1	14.7	8.1		13.4	7.4	<0.001
	**Β**		**β**	**95% CI**	**P**	**Β**	**95% CI**	**P**
Model 1	Reference		−0.98	−1.96 0.00	0.05	−2.64	−3.62 −1.66	<0.001
Model 2	Reference		−0.46	−1.50 0.58	0.38	−1.92	−2.98 −0.86	<0.001

Proanthocyanids	Mean	SD	Mean	SD		Mean	SD	P
2014 CV risk score	28.8	15.4	25.3	12.6		23.8	10.7	<0.001
	**Β**		**β**	**95% CI**	**P**	**Β**	**95% CI**	**P**
Model 1	Reference		−2.39	−3.88 −0.90	0.002	−4.29	−5.78 −2.80	<0.001
Model 2	Reference		−1.07	−2.56 0.42	0.16	−2.60	−4.13 −1.07	<0.001

	Mean	SD	Mean	SD		Mean	SD	P
Changes in CV risk score	16.1	10.2	14.7	8.0		13.3	7.4	<0.001
	**Β**		**β**	**95% CI**	**P**	**Β**	**95% CI**	**P**
Model 1	Reference		−0.96	−1.94 0.02	0.06	−2.65	−3.65 −1.65	<0.001
Model 2	Reference		−0.43	−1.47 0.61	0.41	−1.93	−2.99 −0.87	<0.001

Flavan-3-ols	Mean	SD	Mean	SD		Mean	SD	P
2014 CV risk score	28.1	15.1	25.6	12.7		24.2	11.3	<0.001
	**Β**		**β**	**95% CI**	**P**	**Β**	**95% CI**	**P**
Model 1	Reference		−1.80	−3.29 −0.31	0.02	−3.20	−4.69 −1.71	<0.001
Model 2	Reference		−0.70	−2.17 0.77	0.35	−1.92	−3.41 −0.43	0.01

	Mean	SD	Mean	SD		Mean	SD	P
Changes in CV risk score	15.8	9.8	14.9	8.4		13.6	7.5	<0.001
	**Β**		**β**	**95% CI**	**P**	**Β**	**95% CI**	**P**
Model 1	Reference		−0.58	−1.58 0.42	0.25	−1.94	−2.94 −0.94	<0.001
Model 2	Reference		−0.07	−1.09 0.95	0.89	−1.39	−2.41 −0.37	0.007

Anthocyanidins	Mean	SD	Mean	SD		Mean	SD	P
2014 CV risk score	27.9	14.7	25.5	12.6		24.5	12.0	<0.001
	**Β**		**β**	**95% CI**	**P**	**Β**	**95% CI**	**P**
Model 1	Reference		−1.60	−3.09 −0.11	0.04	−2.73	−4.22 −1.24	<0.001
Model 2	Reference		−0.81	−2.26 0.64	0.28	−1.05	−2.11 0.00	0.05

	Mean	SD	Mean	SD		Mean	SD	P
Changes in CV risk score	15.7	9.4	14.7	8.5		13.8	8.0	0.001
	**Β**		**β**	**95% CI**	**P**	**Β**	**95% CI**	**P**
Model 1	Reference		−0.68	−1.68 0.32	0.18	−1.65	−2.65 −0.65	0.001
Model 2	Reference		−0.27	−1.29 0.75	0.60	−0.90	−1.74 −0.06	0.03

Flavanones	Mean	SD	Mean	SD		Mean	SD	P
2014 CV risk score	28.2	14.8	25.8	12.5		23.9	11.9	<0.001
	**Β**		**Β**	**95% CI**	**P**	**Β**	**95% CI**	**P**
Model 1	Reference		−2.38	−3.87 −0.89	0.002	−3.90	−5.39 −2.41	<0.001
Model 2	Reference		−1.90	−3.35 −0.45	0.01	−2.70	−4.19 −1.21	<0.001

	Mean	SD	Mean	SD		Mean	SD	P
Changes in CV risk score	15.8	9.3	14.7	8.9		13.7	7.7	<0.001
	**Β**		**Β**	**95% CI**	**P**	**Β**	**95% CI**	**P**
Model 1	Reference		−1.13	−2.11 −0.15	0.02	−1.97	−2.95 −0.99	<0.001
Model 2	Reference		−0.98	−1.98 0.02	0.06	−1.51	−2.55 −0.47	0.004

Flavonols	Mean	SD	Mean	SD		Mean	SD	P
2014 CV risk score	27.5	15.0	25.2	12.2		25.2	12.1	0.004
	**Β**		**Β**	**95% CI**	**P**	**Β**	**95% CI**	**P**
Model 1	Reference		−2.10	−3.59 −0.61	0.006	−2.33	−3.82 −0.84	0.002
Model 2	Reference		−1.13	−2.60 0.34	0.13	−1.21	−2.40 −0.02	0.04

	Mean	SD	Mean	SD		Mean	SD	P
Changes in CV risk score	15.4	9.5	14.5	7.9		14.3	8.6	<0.001
	**Β**		**Β**	**95% CI**	**P**	**Β**	**95% CI**	**P**
Model 1	Reference		−0.84	−1.82 0.14	0.10	−1.29	−2.29 −0.29	0.01
Model 2	Reference		−0.44	−1.46 0.58	0.39	−0.72	−1.78 0.34	0.18

Flavones	Mean	SD	Mean	SD		Mean	SD	P
2014 CV risk score	28.2	15.2	25.4	12.3		24.3	11.6	<0.001
	**Β**		**Β**	**95% CI**	**P**	**Β**	**95% CI**	**P**
Model 1	Reference		−2.59	−4.08 −1.10	<0.001	−3.43	−4.92 −1.94	<0.001
Model 2	Reference		−1.74	−3.21 −0.27	0.02	−2.12	−3.65 −0.59	0.007

	Mean	SD	Mean	SD		Mean	SD	P
Changes in CV risk score	15.8	10.1	14.5	8.3		13.9	7.3	0.001
	**Β**		**Β**	**95% CI**	**P**	**Β**	**95% CI**	**P**
Model 1	Reference		−1.37	−2.37 −0.37	0.007	−1.75	−2.75 −0.75	<0.001
Model 2	Reference		−1.02	−2.02 −0.02	0.04	−1.28	−2.32 −0.24	0.02

Isoflavones	Mean	SD	Mean	SD		Mean	SD	P
2014 CV risk score	26.5	13.2	27.1	14.1		24.4	12.2	0.002
	**Β**		**Β**	**95% CI**	**P**	**Β**	**95% CI**	**P**
Model 1	Reference		0.72	−0.77 2.21	0.34	−1.54	−3.03 −0.05	0.04
Model 2	Reference		0.97	−0.46 2.40	0.19	−0.35	−1.82 1.12	0.64

	Mean	SD	Mean	SD		Mean	SD	P
Changes in CV risk score	14.8	8.4	15.4	9.6		14.1	7.9	0.03
	**Β**		**Β**	**95% CI**	**P**	**Β**	**95% CI**	**P**
Model 1	Reference		0.74	−0.25 1.73	0.15	−0.45	−1.45 0.55	0.38
Model 2	Reference		0.96	−0.06 1.98	0.06	0.03	−0.99 1.05	0.96

*Model 1* adjusted for BMI, education, living in a rural area, *Model 2* adjusted for BMI, education, living in a rural area, METS (h/week), alcohol intake, history of CV diseases, values of fasting glucose, log-CRP, fiber, and saturated fatty acid intakes.

During follow-up, 125 incident CV events were diagnosed and 220 deaths occurred, 84 of which due to CV causes (Table 4). The incidence of CV events was significantly lower in individuals with the higher intake of total flavonoids and with higher intake of all subclasses of flavonoids, but flavones and isoflavones, in Cox-regression models after adjustments for age, sex, BMI, education, living in a rural area, METs (hour/week), alcohol, fiber, and saturated fatty acid intakes, smoking, values of systolic and diastolic blood pressure, total and HDL-cholesterol, fasting glucose, CRP, statin and aspirin use. HRs ranged from 0.42 for the higher tertile of flavan-3-ols to 0.56 for the higher tertile of anthocyanidins in the Cox model after multiple adjustments.

**Table 4:** Cardiovascular events and all-cause and cardiovascular mortality by tertiles of flavonoid intake (the first the lower; the third, the higher)

	First tertile	Second tertile			Third tertile		
**Total flavonoids**							
Incident CV events	54	40			31		
	**HR**	**HR**	**95% CI**	**P**	**HR**	**95% CI**	**P**
Model 1	1	0.65	0.42–0.99	0.05	0.45	0.28–0.73	0.001
Model 2	1	0.64	0.42–1.00	0.05	0.46	0.28–0.75	0.002
CV mortality	34	26			24		
	**HR**	**HR**	**95% CI**	**P**	**HR**	**95% CI**	**P**
Model 1	1	0.97	0.56–1.67	0.90	0.81	0.45–1.44	0.47
Model 2	1	0.95	0.54–1.66	0.85	0.83	0.46–1.51	0.55
All-cause mortality	89	69			62		
	**HR**	**HR**	**95% CI**	**P**	**HR**	**95% CI**	**P**
Model 1	1	0.86	0.62–1.21	0.38	0.73	0.51–1.04	0.08
Model 2	1	0.90	0.65–1.26	0.52	0.78	0.55–1.13	0.19
**Proanthocyanids**							
Incident CV events	57	37			31		
	**HR**	**HR**	**95% CI**	**P**	**HR**	**95% CI**	**P**
Model 1	1	0.56	0.36–0.86	0.01	0.42	0.26–0.68	<0.001
Model 2	1	0.56	0.36–0.87	0.009	0.43	0.27–0.70	0.001
CV mortality	34	27			23		
	**HR**	**HR**	**95% CI**	**P**	**HR**	**95% CI**	**P**
Model 1	1	0.99	0.58–1.70	0.97	0.77	0.43–1.39	0.39
Model 2	1	0.98	0.56–1.69	0.93	0.80	0.44–1.46	0.46
All-cause mortality	90	70			60		
	**HR**	**HR**	**95% CI**	**P**	**HR**	**95% CI**	**P**
Model 1	1	0.85	0.61–1.19	0.35	0.69	0.48–0.99	0.05
Model 2	1	0.88	0.63–1.24	0.46	0.75	0.52–1.08	0.12
**Flavan-3-ols**							
Incident CV events	57	42			26		
	**HR**	**HR**	**95% CI**	**P**	**HR**	**95% CI**	**P**
Model 1	1	0.69	0.46–1.05	0.08	0.40	0.25–0.65	<0.001
Model 2	1	0.71	0.47–1.08	0.11	0.42	0.26–0.68	<0.001
CV mortality	37	23			24		
	**HR**	**HR**	**95% CI**	**P**	**HR**	**95% CI**	**P**
Model 1	1	0.75	0.44–1.29	0.30	0.70	0.40–1.20	0.19
Model 2	1	0.79	0.46–1.37	0.40	0.72	0.41–1.26	0.25
All-cause mortality	92	71			57		
	**HR**	**HR**	**95% CI**	**P**	**HR**	**95% CI**	**P**
Model 1	1	0.84	0.61–1.15	0.27	0.63	0.44–0.89	0.009
Model 2	1	0.86	0.62–1.19	0.36	0.68	0.48–0.96	0.03
**Anthocyanidins**							
Incident CV events	53	35			37		
	**HR**	**HR**	**95% CI**	**P**	**HR**	**95% CI**	**P**
Model 1	1	0.59	0.38–0.92	0.02	0.58	0.37–0.92	0.02
Model 2	1	0.58	0.37–0.91	0.02	0.56	0.36–0.89	0.02
CV mortality	40	20			24		
	**HR**	**HR**	**95% CI**	**P**	**HR**	**95% CI**	**P**
Model 1	1	0.58	0.33–1.01	0.05	0.65	0.37–1.15	0.14
Model 2	1	0.56	0.32–0.98	0.04	0.67	0.38–1.18	0.16
All-cause mortality	95	62			63		
	**HR**	**HR**	**95% CI**	**P**	**HR**	**95% CI**	**P**
Model 1	1	0.69	0.50–0.96	0.03	0.66	0.47–0.94	0.02
Model 2	1	0.66	0.47–0.94	0.02	0.66	0.46–0.95	0.02
**Flavanones**							
Incident CV events	54	42			29		
	**HR**	**HR**	**95% CI**	**P**	**HR**	**95% CI**	**P**
Model 1	1	0.71	0.47–1.07	0.11	0.45	0.28–0.73	0.001
Model 2	1	0.73	0.48–1.10	0.13	0.48	0.29–0.77	0.003
CV mortality	39	24			21		
	**HR**	**HR**	**95% CI**	**P**	**HR**	**95% CI**	**P**
Model 1	1	0.67	0.40–1.13	0.14	0.56	0.32–0.99	0.05
Model 2	1	0.71	0.42–1.20	0.20	0.66	0.37–1.17	0.15
All-cause mortality	91	80			49		
	**HR**	**HR**	**95% CI**	**P**	**HR**	**95% CI**	**P**
Model 1	1	0.91	0.67–1.24	0.54	0.54	0.37–0.78	0.001
Model 2	1	0.94	0.68–1.29	0.69	0.59	0.40–0.85	0.005
**Flavonols**							
Incident CV events	56	31			38		
	**HR**	**HR**	**95% CI**	**P**	**HR**	**95% CI**	**P**
Model 1	1	0.49	0.31–0.76	0.002	0.53	0.34–0.83	0.006
Model 2	1	0.51	0.32–0.80	0.003	0.53	0.34–0.83	0.005
CV mortality	36	22			26		
	**HR**	**HR**	**95% CI**	**P**	**HR**	**95% CI**	**P**
Model 1	1	0.63	0.36–1.09	0.10	0.68	0.39–1.19	0.18
Model 2	1	0.69	0.40–1.20	0.19	0.72	0.41–1.27	0.26
All-cause mortality	91	64			65		
	**HR**	**HR**	**95% CI**	**P**	**HR**	**95% CI**	**P**
Model 1	1	0.72	0.51–1.00	0.05	0.70	0.50–0.99	0.05
Model 2	1	0.78	0.55–1.08	0.14	0.72	0.51–1.02	0.06
**Flavones**							
Incident CV events	42	51			32		
	**HR**	**HR**	**95% CI**	**P**	**HR**	**95% CI**	**P**
Model 1	1	1.13	0.74–1.72	0.56	0.68	0.41–1.10	0.11
Model 2	1	1.14	0.75–1.75	0.54	0.66	0.40–1.09	0.10
CV mortality	30	31			23		
	**HR**	**HR**	**95% CI**	**P**	**HR**	**95% CI**	**P**
Model 1	1	1.08	0.64–1.82	0.77	0.87	0.48–1.56	0.63
Model 2	1	1.10	0.65–1.87	0.72	0.83	0.45–1.52	0.55
All-cause mortality	88	71			61		
	**HR**	**HR**	**95% CI**	**P**	**HR**	**95% CI**	**P**
Model 1	1	0.79	0.57–1.09	0.16	0.71	0.50–1.01	0.06
Model 2	1	0.83	0.60–1.16	0.28	0.73	0.51–1.05	0.09
**Isoflavones**							
Incident CV events	48	38			39		
	**HR**	**HR**	**95% CI**	**P**	**HR**	**95% CI**	**P**
Model 1	1	0.78	0.51–1.20	0.26	0.77	0.49–1.19	0.23
Model 2	1	0.81	0.53–1.25	0.35	0.77	0.49–1.21	0.26
CV mortality	30	34			20		
	**HR**	**HR**	**95% CI**	**P**	**HR**	**95% CI**	**P**
Model 1	1	1.23	0.74–2.03	0.42	0.78	0.44–1.41	0.42
Model 2	1	1.21	0.73–2.02	0.48	0.74	0.41–1.36	0.34
All-cause mortality	95	63			62		
	**HR**	**HR**	**95% CI**	**P**	**HR**	**95% CI**	**P**
Model 1	1	0.68	0.49–0.94	0.02	0.70	0.50–0.98	0.04
Model 2	1	1.45	1.05–2.00	0.03	1.39	1.00–1.95	0.05

*Model 1* adjusted for age, sex, BMI, education, living in a rural area, METs (h/week), fiber and saturated fatty acid intakes, *Model 2* adjusted for age, sex, BMI, education, living in a rural area, METs (hour/week), fiber, and saturated fatty acid intakes, alcohol intake, smoking, values of systolic and diastolic blood pressure, total and HDL-cholesterol, fasting glucose, CRP, statin and aspirin use.

Total and subclasses of flavonoids were not significantly associated with the risk of CV mortality in the same Cox model (Table 4).

Being in the third tertile of flavan-3-ols (HR = 0.68; 95% CI 0.48–0.96), anthocyanidins (HR = 0.66; 95% CI 0.46–0.95) and flavanones (HR = 0.59; 95% CI 0.40–0.85) was inversely associated with all-cause mortality.

Data did not change after excluding the 138 under-reporters, the 79 women on estrogen therapy, the 55 individuals on nutritional supplements, and after adjusting for antioxidant vitamin intakes.

## Discussion

The results of this population-based cohort study suggest that higher dietary intakes of flavonoids may be associated with a reduced CV risk score and a 40–50% lower risk of non-fatal CV events.

This is intriguing since in our cohort the consumption of some flavonoid-rich foods inversely associated with CV risk such as cocoa, soybean and tea [13, 17, 28, 29], is infrequent, being fruits and red wine the main sources of flavonoids. Epidemiological studies have suggested that a Mediterranean diet reduces the CV risk [42] and a high concentration of flavonoids has been found in fruits, vegetables, red wine and other elements of the Mediterranean diet. However, there is inconsistent evidence on the role of flavonoids derived from these foods and CV risk, since previous studies reported either a decreased CV incidence and mortality with increased intake of apples, pears, and red wine [8, 12, 16, 24, 25], or no significant effect [6, 10, 21, 26, 27].

We have found both a lower CV risk score at baseline and at follow-up in the higher tertile of flavonoid intake. Intriguingly, the increase in the score from enrolment to the end of follow-up was higher in those individuals. Accordingly, the consumption of flavonoid-rich food has been associated with lower systolic blood pressure [15, 43, 44], lower total cholesterol [44], higher HDL cholesterol values [44–46].

Benefits of flavonoids on blood pressure, lipid values, insulin resistance, and flow-mediated dilatation seem to derive above all from soy, cocoa and tea, as suggested by systematic reviews [47, 48]. However, more recently, flavonoids from fruits and vegetables have been reported to reduce the risk of diabetes mellitus and to improve microvascular reactivity and inflammatory status [49–51]. Accordingly, although a small number of incident CV events occurred in our cohort, the risk of non-fatal CV events was significantly lower in individuals with the higher intake of total and all subclasses of flavonoids, but flavones and isoflavones, which were consumed at negligible concentrations in our cohort. Therefore, the dietary intakes of flavonoids seems relevant for healthy CV outcomes at relatively low concentrations, since most inverse associations with CV risk score and non-fatal CV events appeared with intermediate or low intakes of specific subclasses, suggesting that even small amounts may be beneficial. However, a threshold of intake is probably needed, under which these compounds are unlike to be active.

Flavonoids can inhibit or induce a large variety of enzyme systems, involved in pathways regulating platelet aggregation, inflammatory and immune responses [1, 52, 53]. Furthermore, by their antioxidant properties, flavonoids may protect tissues against oxygen free radicals and lipid peroxidation, thus contributing to the prevention of atherosclerosis, chronic inflammation and cancer [1, 52, 53]. Because of their antioxidant and chelating properties, flavonoids may inactivate reactive oxygen species (ROS) and counteract the oxidation of circulating LDL particles [52–54]. Other anti-atherogenic actions proposed for these compounds are: reduction of the activity of enzymes increasing ROS production; inhibition of HMG-CoA reductase, cholesteryl ester transfer protein (CEPT), angiotensin-converting enzyme, signal transducers and activators of transcription (STAT), and glucose transporters; synthesis of nitric oxide; inhibition of platelet activation and function; anti-angiogenetic effects; improvement in endothelial function, vascular fragility, cellular permeability [54–56]. The anti-inflammatory properties of flavonoids may be due to the inhibition of NF-κB activation and adhesion molecule expression; suppression of the activity and secretion of inflammatory cells; reduction of the concentrations of CRP and cytokines [57, 58].

The associations with fatal events were controversial in our cohort. No significant association was found with CV mortality, probably because the number of fatal CV events was low. Otherwise, many flavonoid subclasses, such as flavan-3-ols, anthocyanidins and flavanones were inversely associated with all-cause mortality. Previous studies have reported a reduced total and/or CV mortality with proanthocyanids [19], flavan-3-ols, [11, 19, 25], anthocyanidins [16, 19], flavonols [13, 19], flavanones [16, 18], flavones [16, 19], and isoflavones [17, 44–47]. On the other hand, other authors reported no protective effects of total or specific subclasses of flavonoids on mortality [7, 12, 21, 22, 27].

These highly divergent results among studies might be due to differences in nutritional, socio-cultural and ethnic characteristics.

The median intakes of flavonoids are highly variable among studies, and values ranging from 50 to 450 mg have been reported in European studies [54]. In particular, the following median intakes have been described for Mediterranean countries: 92 mg/day in Greece [59] and 332.4 mg/day in Spain [60]. On the other hand, in non-Mediterranean countries, the median consumption of flavonoids was much lower, varying from 203 mg/day in US population [19] to 88 mg/day in Sweden, and 13 mg/day in Finland [61]. Our values were between these extreme intakes, in line with other Italian data [62, 63]. The high consumption of red wine and fruits, such as apples and citrus fruits, in our Italian cohort justify the higher intake of total flavonoids and proanthocyanidins with respect to other non-Mediterranean cohorts [19, 61]. On the other hand, the low consumption of tea, justified the lower intakes of flavon-3-ols (in particular epigallocatechin 3-gallate, epicatechin 3-gallate and epigallocatechin) with respect to UK and Ireland [61], and the negligible use of soy explain why the intake of isoflavones and flavones was much lower in our cohort when compared with Asian studies [17].

In most studies, the higher consumption of flavonoids was associated with an overall healthy dietary and metabolic pattern, in line with our results [8, 10–12, 16–21, 25, 26, 49]. Our cohort indeed included individuals with a low level of education, differently from previous studies performed in samples where most participants had at least a high school education [13, 16, 17, 19, 26].

Finally, many compounds tend to be present in the same foods: for example, in our cohort, individuals with lower intakes of flavonoids, ate less fiber and antioxidant vitamins and more saturated fats. It is therefore difficult to ascertain the independent effect of dietary components because of multicollinearity. However, our associations remained significant after adjusting for these dietary factors, thus suggesting that a higher flavonoid intake might not merely be an indicator of a healthier lifestyle.

### Limitations

The EPIC questionnaire was not originally designed to measure flavonoid intake, but it has been extensively used and validated for this purpose [60, 64, 65].

The flavonoid intake might have been underestimated because of the limitations of the food composition databases. It should be noted that the presence of particular flavonoids in vegetables and fruits depends on the crop variety, location and type of cultivation. The adaptability of the USDA database to the Italian diet is questionable. The absorption and microbial transformation in the gut of specific subclasses of flavonoids vary considerably, therefore the different flavonoid bioavailability could have an impact on the associations between the assumption of these compounds and chronic diseases. In general, flavonoid subclasses are present simultaneously in foods and establishing which of the compound is responsible for the potential biological effect is difficult. We relied on dietary intake from the questionnaire administered at one point in time; thus misclassification of dietary exposure might have occurred if individuals have changed their diets during the follow-up. Furthermore, measurement error in collecting self-reported dietary intake is inevitable and our observational study was prone to the possibility of unmeasured confounding.

However, the recent versions of the USDA database includes more cooked foods [2], because in culinary preparations important losses in flavonoid content occur, and is the most complete and used database in the estimation of flavonoid intake. Moreover, we have referred also to a European database, and the USDA has been already used for the Italian population [62–64]. We have used a validated instrument and, both at baseline and at follow-up, the associations between flavonoid intakes and the CV risk score were consistent. Measurement errors and misclassification was likely to be random and would have attenuated the association found. We have took care to adjust for many potential confounders. Finally, we have studied a large population-based cohort from a localized region, with a high level of participation, which could have limited the number of potential confounders.

## Conclusions

Individuals with higher intakes of flavonoids showed a lower CV risk after a mean 12-year follow-up, and a reduced risk of non-fatal CV events. If these results will be confirmed in larger prospective cohorts, it would be useful to obtain reliable markers of flavonoid intake in order to define the optimal doses of specific flavonoids for CV protection.

## Abbreviations

BMI: body mass index
CEPT: cholesteryl ester transfer protein
CI: confidence intervals
CRP: high-sensitivity C-reactive protein
CV: cardiovascular
HR: hazard ratio
ICD: International Classification of Diseases
ROS: reactive oxygen species
STAT: signal transducers and activators of transcription

## References

[1] Hollman PCH, Katan MB (1999). Dietary flavonoids: intake, health effects and bioavailability. Food Chem Toxicol.

[2] Agricultural Research Service. US Department of Agriculture (USDA) (2014) Database for the flavonoid content of selected foods, Beltsville. http://www.ars.usda.gov/SP2UserFiles/Place/80400525/Data/Flav/Flav_R03-1.pdf. Accessed 5 Jun 2015

[3] Agricultural Research Service. US Department of Agriculture (USDA) (2008) Database for the isoflavone content of selected foods, Beltsville. http://www.ars.usda.gov/SP2UserFiles/Place/80400525/Data/isoflav/Isoflav_R2.pdf. Accessed 9 Jun 2015

[4] Agricultural Research Service. US Department of Agriculture (USDA) (2004) Database for the proanthocyanidin content of selected foods, Beltsville. http://www.ars.usda.gov/SP2UserFiles/Place/80400525/Data/PA/PA.pdf. Accessed 7 Jun 2015

[5] Middleton E, Kandaswami C, Theoharides TC (2000). The effects of plant flavonoids on mammalian cells: implications for inflammation, heart disease, and cancer. Pharmacol Rev.

[6] Hertog MGL, Feskens EJM, Hollman PCH, Katan MB, Kromhout D (1993). Dietary antioxidant flavonoids and risk of coronary heart disease: the Zutphen Elderly Study. Lancet.

[7] Hertog MG, Kromhout D, Aravanis C, Blackburn H, Buzina R, Fidanza F (1995). Flavonoid intake and long-term risk of coronary heart disease and cancer in the seven countries study. Arch Intern Med.

[8] Knekt P, Järvinen R, Reunanen A, Maatela J (1996). Flavonoid intake and coronary mortality in Finland: a cohort study. Br Med J.

[9] Hertog MG, Feskens EJM, Kromhout D (1997). Antioxidant flavonols and coronary heart disease risk. Lancet.

[10] Yochum L, Kushi LH, Meyer K, Folsom AR (1999). Dietary flavonoid intake and risk of cardiovascular disease in postmenopausal women. Am J Epidemiol.

[11] Arts IC, Hollman PCH, Feskens EJM, de Mesquita HBB, Kromhout D (2001). Cathechin intake might explain the inverse relation between tea consumption and ischemic heart disease: the Zutphen Elderly Study. Am J Clin Nutr.

[12] Hirvonen T, Pietinen P, Virtanen M, Ovaskainen ML, Häkkinen S, Albanes D (2001). Intake of flavonols and flavones and risk of coronary heart disease in male smokers. Epidemiology.

[13] Geleijnse JM, Launer LJ, van der Kuip DAM, Hofman A, Witteman JCM (2002). Inverse association of tea and flavonoid intakes with incident myocardial infarction: the Rotterdam Study. Am J Clin Nutr.

[14] Knekt P, Kumpulainen J, Järvinen R, Rissanen H, Heliövaara M, Reunanen A (2002). Flavonoid intake and risk of chronic diseases. Am J Clin Nutr.

[15] Mennen LI, Sapinho D, de Bree A, Arnault N, Bertrais S, Galan P (2004). Consumption of foods rich in flavonoids is related to a decreased cardiovascular risk in apparently healthy French women. J Nutr.

[16] Mink PJ, Scrafford CG, Barraj LM, Harnack L, Hong CP, Nettleton JA (2007). Flavonoid intake and cardiovascular disease mortality: a prospective study in postmenopausal women. Am J Clin Nutr.

[17] Kokubo Y, Iso H, Ishihara J, Okada K, Inoue M, Tsugane S, for the JPHC Study Group (2007). Association of dietary intake of soy, beans, and isoflavones with risk of cerebral and myocardial infarctions in Japanese populations. Circulation.

[18] Mursu J, Voutilainen S, Nurmi T, Tuomainen TP, Kurl S, Salonen JT (2008). Flavonoid intake and the risk of ischaemic stroke and CVD mortality in middle-aged Finnish men: the Kuopio Ischaemic Heart Disease Risk Factor Study. Br J Nutr.

[19] McCullough ML, Peterson JJ, Patel R, Jacques PF, Shah R, Dwyer JT (2012). Flavonoid intake and cardiovascular disease mortality in a prospective cohort of US adults. Am J Clin Nutr.

[20] Cassidy A, Mukamal KJ, Liu L, Franz M, Eliassen AH, Rimm EB (2013). High anthocyanin intake is associated with a reduced risk in myocardial infarction in young and middle-aged women. Circulation.

[21] Rimm EB, Katan MB, Ascherio A, Stampfer MJ, Willett WC (1996). Relation between intake of flavonoids and risk for coronary heart disease in male health professionals. Ann Intern Med.

[22] Hertog MGL, Sweetnam PM, Fehily AM, Elwood PC, Kromhout D (1997). Antioxidant flavonols and ischemic heart disease in a Welsh population of men: the Caerphilly Study. Am J Clin Nutr.

[23] Hirvonen T, Virtamo J, Korhonen P, Albanes D, Pietinen P (2000). Intake of flavonoids, carotenoids, vitamins C and E, and risk of stroke in male smokers. Stroke.

[24] Knekt P, Isotupa S, Rissanen H, Heliövaara M, Järvinen R, Häkkinen S (2000). Quercetin intake and the incidence of cerebrovascular disease. Eur J Clin Nutr.

[25] Arts ICW, Jacobs DR, Harnack LJ, Gross M, Folsom AR (2001). Dietary catechins in relation to coronary heart disease death among postmenopausal women. Epidemiology.

[26] Sesso HD, Gaziano JM, Liu S, Buring JE (2003). Flavonoid intake and the risk of cardiovascular disease in women. Am J Clin Nutr.

[27] Lin J, Rexrode KM, Hu F, Albert CM, Chae CU, Rimm EB (2007). Dietary intakes of flavonols and flavones and coronary heart disease in US women. Am J Epidemiol.

[28] Hooper L, Kroon PA, Rimm EB, Cohn JS, Harvey I, Le Cornu CA (2008). Flavonoids, flavonoid-rich foods, and cardiovascular risk: a meta-analysis of randomized controlled trails. Am J Clin Nutr.

[29] Keli SO, Hertog MGL, Feskens EJM, Kromhout D (1996). Dietary flavonoids, antioxidant vitamins, and incidence of stroke. The Zutphen study. Arch Intern Med..

[30] Hooper L, Kay C, Abdelhamid A, Kroon PA, Cohn JS, Rimm EB (2012). Effects of chocolate, cocoa, and flavan-3-ols on cardiovascular health: a systematic review and meta-analysis of randomized trials. Am J Clin Nutr.

[31] Bo S, Gentile L, Ciccone G, Baldi C, Benini L, Dusio F (2005). The metabolic syndrome and high C-reactive protein: prevalence and difference by sex in a southern-European population-based cohort. Diabetes Metab Research Rev.

[32] Taylor HL, Jacobs DR, Schucker B, Knudsen J, Leon AS, Debacker G (1978). Questionnaire for the assessment of leisure time physical activities. J Chronic Diseases.

[33] Bo S, Durazzo M, Guidi S, Carello M, Sacerdote C, Silli B (2006). Dietary magnesium and fiber intake, inflammatory and metabolic parameters in middle-aged subjects from a population-based cohort. Am J Clin Nutr.

[34] Kroke A, Klipstein-Grobusch K, Voss S, Moseneder J, Thielecke F, Noack R (1999). Validation of a self-administered food-frequency questionnaire administered in the European Prospective Investigation into Cancer and Nutrition (EPIC) study: comparison of energy, protein, and macronutrient intakes estimated with the doubly labeled water, urinary nitrogen, and repeated 24-h dietary recall methods. Am J Clin Nutr.

[35] Willett W, Stampfer MJ (1986). Total energy intake: implications for epidemiologic analyses. Am J Epidemiol.

[36] Schofield WN (1985). Predicting basal metabolic rate, new standards and review of previous work. Hum Nutr Clin Nutr.

[37] Goldberg GR, Black AE, Jebb SA, Cole TJ, Murgatroyd PR, Coward WA (1991). Critical evaluation of energy intake data using fundamental principles of energy physiology: 1. derivation of cut-off limits to identify under-recording. Eur J Clin Nutr.

[38] Gry J, Black L, Eriksen FD, Pilegaard K, Plumb J, Rhodes M (2007). EuroFIR-BASIS—a combined composition and biological activity database for bioactive compounds in plant-based foods. Trends Food Sci Tech.

[39] Istituto Nazionale di Ricerca per gli Alimenti e la Nutrizione (INRAN) (2003) Linee guida per una sana alimentazione italiana. http://www.piramidealimentare.it/files_allegati/guida.pdf. Accessed 5 Jun 2015 **(in Italian)**

[40] American Diabetes Association (2014). Diagnosis and classification of diabetes mellitus. Diabetes Care.

[41] D’Agostino RB, Vasan RS, Pencina MJ, Wolf PA, Cobain M, Massaro JM (2008). General cardiovascular risk profile for use in primari care: the Framingham Heart Study. Circulation.

[42] Grosso G, Mistretta A, Frigiola A, Gruttadauria S, Biondi A, Basile F (2014). Mediterranean diet and cardiovascular risk factors: a systematic review. Crit Rev Food Sci Nutr.

[43] Cassidy A, O’Reilly EJ, Kay C, Sampson L, Franz M, Forman JP (2011). Habitual intake of flavonoid subclasses and incident hypertension in adults. Am J Clin Nutr.

[44] Sagara M, Kanda T, Jelekera MN, Teramoto T, Armitage L, Birt N (2003). Effects of dietary intake of soy protein and isoflavones on cardiovascular disease risk factors in high risk, middle-aged men in Scotland. J Am Coll Nutr.

[45] Kurowska EM, Spence JD, Jordan J, Wetmore S, Freeman DJ, Pinché LA (2000). HDL-cholesterol-raising effect of orange juice in subjects with hypercholesterolemia. Am J Clin Nutr.

[46] Qin Y, Xia M, Ma J, Hao JT, Liu J, Mou H (2009). Anthocyanin supplementation improves serum LDL- and HDL-cholesterol concentrations associated with the inhibition of cholesteryl ester transfer protein in dyslipidemic subjects. Am J Clin Nutr.

[47] Hooper L, Kroom PA, Rimm EB, Cohn JS, Harvey I, Le Cornu KA (2008). Flavonoids, flavonoid-rich foods, and cardiovascular risk: a meta-analysis of randomized controlled trials. Am J Clin Nutr.

[48] Hooper L, Key C, Abdelhamid A, Kroom PA, Cohn JS, Rimm EB (2012). Effects of chocolate, cocoa and flavan-3-ols on cardiovascular health: a systematic review and meta-analysis of randomized trials. Am J Clin Nutr.

[49] Wedick NM, Pan A, Cassidy A, Rimm EB, Sampson L, Rosner B (2012). Dietary flavonoids intakes and risk of type 2 diabetes in US men and women. Am J Clin Nutr.

[50] Jennings A, Welch AA, Fairweather-Tait SJ, Kay C, Minihane AM, Chowienczyk P (2012). Higher anthocyanin intake is associated with lower arterial stiffness and central blood pressure in women. Am J Clin Nutr.

[51] Macready AL, George TW, Chong MF, Alimbertov DS, Jin Y, Vidal A (2014). Flavonoid-rich fruit and vegetables improve microvascular reactivity and inflammatory status in men at risk of cardiovascular disease-FLAVURS: a randomized controlled trial. Am J Clin Nutr.

[52] De Pascual-Teresa S, Moreno DA, García-Viguera C (2010). Flavanols and anthocyanins in cardiovascular health: a review of current evidence. Int J Mol Sci.

[53] Middleton E, Kandaswami C, Theoharides TC (2000). The effects of plant flavonoids on mammalian cells: implications for inflammation, heart disease, and cancer. Pharmacol Rev.

[54] Kozkowska A, Szostak-Węgierek D (2014). Flavonoids—food sources and health benefits. Rocz Państw Zakł Hig.

[55] Rein D, Paglieroni TG, Wun T, Pearson DA, Schmitz HH, Gosselin R (2000). Cocoa inhibits platelet activation and function. Am J Clin Nutr.

[56] Vita JA (2005). Polyphenols and cardiovascular disease: effects on endothelial and platelet function. Am J Clin Nutr.

[57] Landberg R, Sun Q, Rimm EB, Cassidy A, Scalbert A, Mantzoros CS (2011). Selected dietary flavonoids are associated with markers of inflammation and endothelial dysfunction in US women. J Nutr.

[58] Chun OK, Chung SJ, Claycombe KJ, Song WO (2008). Serum C-reactive protein concentrations are inversely associated with dietary flavonoid intake in US adults. J Nutr.

[59] Dilis V, Trichopoulou A (2010). Antioxidant intakes and food sources in Greek adults. J Nutr.

[60] Zamora-Ros R, Sacerdote C, Ricceri F, Weiderpass E, Roswall N, Buckland G (2014). Flavonoid and lignan intake in relation to bladder cancer risk in the European Prospective Investigation into Cancer and Nutrition (EPIC) study. Br J Cancer.

[61] Vogiatzoglou A, Mulligan AA, Lentjes MA, Luben RN, Spencer JP, Schroeter H (2015). Flavonoid intake in European adults (18 to 64 years). PLoS One.

[62] Rossi M, Negri E, Lagiou P, Talamini R, Del Maso L, Montella M (2008). Flavonoids and ovarian cancer: a case-control study in Italy. Int J Cancer.

[63] Società Italiana di Nutrizione Umana (2014) Livelli di assunzione di riferimento di nutrienti ed energia per la popolazione italiana. SICS Ed, October 2014, 4th Revision

[64] Bosetti C, Rossi M, McLaughlin JK, Negri E, Talamini R, Lagiou P (2007). Flavonoids and the risk of renal cell carcinoma. Cancer Epidemiol Biomarkers Prev.

[65] Zamora-Ros R, Agudo A, Luján-Barroso L, Romieu I, Ferrari P, Knaze V (2012). Dietary flavonoid and lignin intake and gastric adenocarcinoma risk in the European Prospective Investigation into Cancer and Nutrition (EPIC) study. Am J Clin Nutr.

